# No-Signaling-in-Time (NSIT) Condition for Energy

**DOI:** 10.3390/e21111067

**Published:** 2019-10-31

**Authors:** Yuxia Zhang, Jian Zou, Bin Shao

**Affiliations:** School of Physics, Beijing Institute of Technology, Beijing 100081, Chinasbin610@bit.edu.cn (B.S.)

**Keywords:** no-signaling-in-time conditions for conditional energy, no-signaling-in-time conditions for average energy, no-signaling-in-time conditions

## Abstract

In this paper, analogous to the no-signaling-in-time (NSIT) conditions, a series of equalities for the change of conditional and average energy of a quantum system are given to test macrorealism. These equalities are named no-signaling-in-time conditions for conditional energy (CNSIT) and no-signaling-in-time conditions for average energy (ANSIT), respectively. Then, we investigate the violations of the NSIT conditions, the CNSIT conditions and the ANSIT conditions for a qubit in the following scenarios: pure coherent dynamics, dynamics with drive, dynamics under dissipation and dephasing. For the pure qubit, when the NSIT conditions or the CNSIT conditions are not violated, the ANSIT conditions can not be violated, and a suitable conjunction of the CNSIT conditions and the NSIT conditions may be better for testing macrorealism. While for the driven qubit, the non-violation of the CNSIT conditions implies the non-violation of the NSIT conditions, which in turn implies the non-violation of the ANSIT conditions. For dephasing and dissipative qubits, the relationships among the NSIT conditions, the CNSIT conditions and the ANSIT conditions are similar to those of the pure and driven qubits, respectively. While the degree of violations of the NSIT conditions, the CNSIT conditions and the ANSIT conditions is decreased with the increasing time interval between measurements; and if this time interval tends to a very large number, all three kinds of conditions are satisfied.

## 1. Introduction

Conceptually and mathematically, quantum physics is incompatible with a view of classical world. Contrary to our classical intuition, the quantum superposition principle describes that an object can be in two different states simultaneously. And how macroscopic classical world emerges from the framework of quantum mechanics has always been a topic of foundational interest. In order to distinguish between classical and quantum behavior, two fundamental concepts for classical physics have been established: local realism [1] and macroscopic realism (or macrorealism) [2]. The local realism, which limits the power of classical experiments to establish correlations over space, has been formulated in the form of the well known Bell inequality [1]. The other fundamental concept, macrorealism, was proposed by Leggett and Garg in 1985, and can be described as [2]: (1) Macrorealism per se (MRps): A macroscopic object which has two or more macroscopically distinct states is in a definite one of those states at any given time; (2) Non-invasive measurability (NIM): In principle, it is possible to determine which of these states the system is in without any influence on the state itself or on the subsequent system dynamics. Based on these assumptions and analogous to Bell’s theorem [1], the Leggett–Garg inequality [2,3,4] was proposed to test quantum correlations in time by Leggett and Garg. Since then, a number of experiments for violations of Leggett–Garg inequality have been performed, and the quantum behaviour can be now confirmed experimentally [5,6,7,8,9,10]. The spatial Bell inequality tests entanglement between spatially-separated systems, while the Leggett–Garg inequality probes the correlations of a single system measured at different times. In addition, the Bell inequality places bound on correlations between measurements for the spatially-separated systems, and the Leggett–Garg inequality places bound on the separation between measurements in time. Therefore, the Leggett–Garg inequality is often referred to as the temporal Bell inequality [11].

Recently, no-signaling-in-time (NSIT) condition has been proposed as another criteria to test macrorealism [12,13,14,15], i.e., to test incompatibility between the classical world view of macrorealism and quantum mechanics. And the NSIT condition is considered as a better candidate for testing macrorealism than the Leggett–Garg inequality [13,14,16], and the former usually can be violated for a much wider parameter regime than the later. Local realism implies the Bell inequality and the no-signaling (NS) condition. The NS condition ensures that probabilities of outcomes for one party must be independent of the setting of the other party in case the relevant events are spacelike separated. The NSIT condition is analogous to the traditional NS condition of the Bell scenario, while it ensures that a measurement does not change the outcome statistics of a later measurement, i.e., it assumes that the probability of obtaining an outcome for subsequent measurements, can not be affected by the prior measurements. And it is obeyed by all macrorealistic theories, and can be regarded as a statistical version of one of macrorealism assumptions, i.e., NIM. When one of the NSIT conditions is violated, macrorealism is violated, i.e., the subsequent measurement is invaded by the relevant prior measurement.

Measurement plays a significant role in quantum mechanics, for example, it can produce an unavoidable stochastic change of system state, and thus modify the quantities of many physical observables. In the framework of physics, there are many physical observables, among which energy is one of the most important. When the system is measured, its energy content may change. And in Reference [17], Chanda et al. investigated the average energy cost of the process associated with the Leggett–Garg inequality, and showed that the maximal violation of Leggett–Garg inequality in the energy constraint, is realized, when the average energy of this process equals to the negative of the energy of the initial state, in noiseless and in certain noisy scenarios. In fact, the NSIT condition takes the measurement probability as a tool to test macrorealism. Here, we are interested in the conditions satisfying macrorealism for energy change in the same scenarios as that of original NSIT conditions. When a measurement is performed, the system may undergo different trajectories, and energy may become trajectory dependent and stochastic [18,19,20]. The average energy change has been well understood, which is simply given by measuring the difference between the average energy of the system before and after the measurement. However, there was a lack of a universal answer to quantify the energy change conditioned on observing a given measurement result until Mohammady et al. [21] provided a definition of conditional energy change. The conditional energy change introduced by Mohammady et al. [21], is the energy change of system before and after the measurement process for a specific outcome, which is suitable for general quantum measurements and arbitrary initial state of system.

In this paper, we want to use the conditional energy change and average energy change to test macrorealism. Analogous to the NSIT conditions, a series of equalities for the conditional and average energy changes are given to test macrorealism, which are called no-signaling-in-time conditions for conditional energy (CNSIT) and no-signaling-in-time conditions for average energy (ANSIT), respectively. When one of the CNSIT conditions or the ANSIT conditions is violated, NIM is violated, so macrorealism can not be satisfied. We consider a qubit in the following scenarios: pure coherent dynamics, dynamics with drive, dynamics under dissipation and dephasing. And two kinds of initial states are considered: mixed and pure states. In the case of coherent dynamics, for both initial mixed and pure states, the non-violation of the ANSIT conditions implies the non-violation of the NSIT conditions and the CNSIT conditions. And the non-violation conditions of the NSIT conditions and the CNSIT conditions do not contain each other, i.e., a suitable conjunction of the CNSIT conditions and the NSIT conditions is tighter for testing macrorealism. Next, for coherent dynamics with drive, in the case of the initial mixed and pure states, when the CNSIT conditions are not violated, the NSIT conditions must not be violated; and when the NSIT conditions are not violated, the ANSIT conditions must not be violated. Next, for both initial mixed and pure states, the relationship among the NSIT conditions, the CNSIT conditions and the ANSIT conditions for the dissipative qubit is similar to that of the driven qubit; and for the dephasing qubit, the relationship is similar to that of the pure qubit. While for dissipative and dephasing qubits, the dissipation and dephasing decrease the degree of violations of the NSIT conditions, the CNSIT conditions and the ANSIT conditions, with the increasing time interval between measurements; and if this time interval tends to very large values, all three kinds of conditions can not be violated.

## 2. NSIT Conditions, CNSIT Conditions and ANSIT Conditions

### 2.1. Conditional Energy Change

Let us briefly review the concept of conditional energy change. The conditional energy change was introduced by Mohammady et al. [21], who described the energy change of the system before and after the measurement process when a specific measurement outcome is given. And it is suitable for general quantum measurements and arbitrary initial state. Consider an observable *Q* of the system. The conditional energy change of the system before and after the measurement process at *t* for a given measurement outcome *n*, $△E^{n}\left(t\right)$, can be described as

(1) △En(t)=Tr[H(t)ρ′(t)]−Re{Tr[Qn(t)H(t)ρ(t)]}P(Qn(t)),

where H(t) and Q(t) are the Hamiltonian and observable of the system at *t*, respectively. Here, ρ(t) and $\rho^{\prime }\left(t\right)$ are the pre-measurement state and the post-measurement state at *t*, respectively, $\rho^{\prime }\left(t\right)=Q^{n}\left(t\right)\rho \left(t\right)Q^{n}{\left(t\right)}^{†}/P\left(Q^{n}\left(t\right)\right)$, and $P\left(Q^{n}\left(t\right)\right)=\mathrm{Tr}\left[Q^{n}\left(t\right)\rho \left(t\right)Q^{n}{\left(t\right)}^{†}\right]$ is the probability of obtaining outcome *n* by measuring at *t*. This definition is different from the previous way of calculating energy change before and after the measurements, i.e., $△E\left(t\right)=\mathrm{Tr}\left[H\left(t\right)\rho^{\prime }\left(t\right)\right]-\mathrm{Tr}\left[H\left(t\right)\rho \left(t\right)\right]$ which has been successfully used in some instances [22,23,24,25,26,27,28], but breaks down sometimes even in a classical probabilistic process [21]. The difference between them is that the conditional energy change quantifies the change of energy conditioned on observing a given measurement outcome. And the conditional energy change (Equation (1)) uses the conditional energy of the initial state for a given measurement outcome, instead of the unconditional one, Tr[H(t)ρ(t)]. It is noted that the increase of average conditional energy equals to the increase of average energy, i.e., ∑nP(Qn(t))Re{Tr[Qn(t)H(t)ρ(t)]}P(Qn(t))=Tr[H(t)ρ(t)]. In other words, we multiply the change of conditional energy (Equation (1)) by its probability, sum all results, and then obtain the corresponding change of average energy $△\bar{E}\left(t\right)$. And this average energy change equals to the corresponding energy change before and after a blind measurement at *t*, $△E_{blind}\left(t\right)$, which can be expressed as

(2) $$△E_{blind}\left(t\right)=\mathrm{Tr}\left[H\left(t\right)\left(\Sigma_{n}Q^{n}\left(t\right)\rho \left(t\right)Q^{n†}\left(t\right)\right)\right]-\mathrm{Tr}\left[H\left(t\right)\rho \left(t\right)\right].$$

Therefore, $\sum_{n}P\left(Q^{n}\left(t\right)\right)△E^{n}\left(t\right)=△\bar{E}\left(t\right)=△E_{blind}^{n}\left(t\right)$.

### 2.2. NSIT Conditions

Now, we briefly review the NSIT conditions [12,13,14,15], which provide a useful tool for testing macrorealism. The two-time ${\mathrm{NSIT}}_{(i)j}^{(n)m}$ conditions, can be expressed as

(3) $${\mathrm{NSIT}}_{(i)j}^{(n)m}:P\left(Q^{m}\left(t_{j}\right)\right)-\sum_{n}P\left(Q^{n}\left(t_{i}\right),Q^{m}\left(t_{j}\right)\right)=0.$$

Here, i=0,1,2 and j=1,2 with i<j, *m* and *n* are outcomes of the observable, $P(Q^{m}\left(t_{j}\right))$ is the probability of obtaining outcome *m* by measuring at $t_{j}$, and $P(Q^{n}\left(t_{i}\right),Q^{m}\left(t_{j}\right))$ is the probability of obtaining outcomes *n* and *m* by measuring at $t_{i}$ and $t_{j}$, respectively. In fact, the two-time NSIT conditions compare the probability of a single observable measured at one time step ($P(Q^{m}\left(t_{j}\right))$) with that measured at two time steps ($P(Q^{n}\left(t_{i}\right),Q^{m}\left(t_{j}\right))$). And the two-time NSIT conditions are satisfied when by measuring $Q(t_{j})$, we can not detect whether a measurement of $Q(t_{i})$ has been performed, i.e., the measurement of $Q(t_{i})$ is not disturbing the statistics of $Q(t_{j})$.

Then, similarly, the three-time conditions ${\mathrm{NSIT}}_{(0)1,2}^{(n)m,l}$ and ${\mathrm{NSIT}}_{0(1)2}^{n(m)l}$ are respectively given by

(4) $$\begin{array}{ccc} {\mathrm{NSIT}}_{(0)1,2}^{(n)m,l} & : & P\left(Q^{m}\left(t_{1}\right),Q^{l}\left(t_{2}\right)\right)-\sum_{n}P\left(Q^{n}\left(t_{0}\right),Q^{m}\left(t_{1}\right),Q^{l}\left(t_{2}\right)\right)=0, \end{array}$$

(5) $$\begin{array}{ccc} {\mathrm{NSIT}}_{0(1)2}^{n(m)l} & : & P\left(Q^{n}\left(t_{0}\right),Q^{l}\left(t_{2}\right)\right)-\sum_{m}P\left(Q^{n}\left(t_{0}\right),Q^{m}\left(t_{1}\right),Q^{l}\left(t_{2}\right)\right)=0, \end{array}$$

where similar to *m* and *n*, *l* is also the outcome of observable, and $P(Q^{n}\left(t_{0}\right),Q^{m}\left(t_{1}\right),Q^{l}\left(t_{2}\right))$ is the probability of obtaining outcomes *m*, *n* and *l* at $t_{0}$, $t_{1}$ and $t_{2}$, respectively. ${\mathrm{NSIT}}_{(0)1,2}^{(n)m,l}$ denotes that the probability of obtaining measurement outcomes at $t_{1}$ and $t_{2}$, is not affected by the prior measurement at $t_{0}$, and similarly for ${\mathrm{NSIT}}_{0(1)2}^{n(m)l}$. In other words, if the relevant prior measurement invades the subsequent measurement, a NSIT condition can be violated [29]. And it is noted that when one of the NSIT conditions is violated, macrorealism is violated.

### 2.3. CNSIT Conditions

As we know, the conjunction of all the NSIT conditions ensures the existence of a global joint probability distribution. In fact, the NSIT condition tests macrorealism by whether the probability of obtaining an outcome of measurement is affected by the prior measurement or not. Similar to the probability of measurement, measurement may modify physical quantities. When the system is measured, its energy content may change. In this paper, we are interested in the conditions of satisfying macrorealism for energy change. Therefore, we want to investigate whether the energy change is independent of that any prior measurement has or has not been performed. According to NIM, we use energy, i.e., the energy change of the whole process, instead of the measurement probability to test macrorealism. Therefore, analogous to the NSIT conditions, a set of equalities for the change of energy is given. We consider the conditional energy change and average energy change, i.e., energy change of a trajectory for a given measurement outcome and an average energy change of different trajectories, to test macrorealism. And when one of the ANSIT conditions or the CNSIT conditions is violated, NIM is violated, and macrorealism is violated.

Analogous to NSIT conditions, the two-time and three-time CNSIT conditions can be expressed as

(6) $$△E_{(i)j}^{(n)m}:△E_{j}^{m}-\sum_{n}△E_{i,j}^{n,m}=0,$$

(7) $$△E_{(0)1,2}^{(n)m,l}:△E_{1,2}^{m,l}-\sum_{n}△E_{0,1,2}^{n,m,l}=0,$$

(8) $$△E_{0(1),2}^{n(m),l}:△E_{0,2}^{n,l}-\sum_{m}△E_{0,1,2}^{n,m,l}=0.$$

For simplicity, we take $△E_{(0)1,2}^{(n)m,l}$ (Equation (7)) as an example to illustrate the CNSIT conditions. Here, $△E_{1,2}^{m,l}$ in Equation (7) is the energy change of the system in the whole process, when a single observable is measured at two time steps. And the energy change of the whole process $△E_{1,2}^{m,l}$ contains:

I. the energy change during the evolution from t=0 to $t=t_{1}$, $△E_{evo}\left(0\to t_{1}\right)$;

II. the conditional energy change $△E^{m}\left(t_{1}\right)$, which is the difference of energy before and after the measurement at $t=t_{1}$ given outcome *m*;

III. the energy change during the evolution from $t=t_{1}$ to $t=t_{2}$, $△E_{evo}^{m}\left(t_{1}\to t_{2}\right)$;

IV. the conditional energy change before and after the measurement at $t=t_{2}$ given outcome *l*, $△E^{m,l}\left(t_{1},t_{2}\right)$.

**Figure 1:**
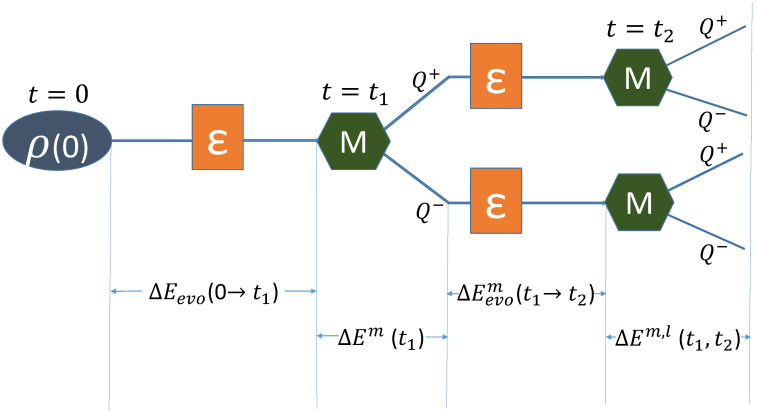
Schematic diagram for the energy change $△E_{1,2}^{m,l}$. Here, we take a general dichotomic measurements $Q^{\pm }$ as an example with m,l=±1 being the measurement outcomes, and ε is the system propagator ρ→ε(ρ).

Thus, $△E_{1,2}^{m,l}=△E_{evo}\left(0\to t_{1}\right)+△E^{m}\left(t_{1}\right)+△E_{evo}^{m}\left(t_{1}\to t_{2}\right)+△E^{m,l}\left(t_{1},t_{2}\right)$, which can be seen from Figure 1. Here, the change of energy during the evolution from $t=t_{i}$ to $t=t_{j}$ can be written as

(9) $$△E_{evo}\left(t_{i}\to t_{j}\right)=\mathrm{Tr}\left[H\left(t_{j}\right)\rho \left(t_{j}\right)\right]-\mathrm{Tr}\left[H\left(t_{i}\right)\rho \left(t_{i}\right)\right],$$

where $H(t_{i})$ and $H(t_{j})$ are Hamiltonians at $t=t_{i}$ and $t=t_{j}$, respectively, $\rho (t_{i})$ is the density matrix at $t=t_{i}$ and $\rho (t_{j})$ is the density matrix at $t=t_{j}$ which is evolved from $\rho (t_{i})$. Then, $△E_{evo}\left(0\to t_{1}\right)$ and $△E_{evo}^{m}\left(t_{1}\to t_{2}\right)$ can be obtained from Equation (9), $△E^{m}\left(t_{1}\right)$ and $△E^{m,l}\left(t_{1},t_{2}\right)$ can be derived from Equation (1).

Similarly, $\sum_{n}△E_{0,1,2}^{n,m,l}$ in $△E_{(0)1,2}^{(n)m,l}$ (Equation (7)) is the energy change of the whole process, when a single observable is measured at three time steps. The energy change of the whole process $\sum_{n}△E_{0,1,2}^{n,m,l}$ of Equation (7) includes:

I. the change of energy during the evolution from t=0 to $t=t_{0}$, $△E_{evo}\left(0\to t_{0}\right)$;

II. the change of energy before and after blind measurement at $t=t_{0}$, $△E_{blind}\left(t_{0}\right)$;

III. the change of energy during the evolution from $t=t_{0}$ to $t=t_{1}$, $△E_{evo}\left(t_{0}\to t_{1}\right)$;

IV. the change of conditional energy before and after measurement at $t=t_{1}$ given outcome *m*, $△E^{m}\left(t_{0},t_{1}\right)$;

V. the change of energy during the evolution from $t=t_{1}$ to $t=t_{2}$, $△E_{evo}^{m}\left(t_{1}\to t_{2}\right)$;

VI. the change of conditional energy $△E^{m,l}\left(t_{0},t_{1},t_{2}\right)$, which is the difference of energy before and after the measurements at $t=t_{2}$ given outcome *l*.

Thus, $\sum_{n}△E_{0,1,2}^{n,m,l}=△E_{evo}\left(0\to t_{0}\right)+△E_{blind}\left(t_{0}\right)+△E_{evo}\left(t_{0}\to t_{1}\right)+△E^{m}\left(t_{0},t_{1}\right)+△E_{evo}^{m}\left(t_{1}\to t_{2}\right)+△E^{m,l}\left(t_{0},t_{1},t_{2}\right)$. Here, $△E_{evo}\left(0\to t_{0}\right)$, $△E_{evo}\left(t_{0}\to t_{1}\right)$ and $△E_{evo}\left(t_{1}\to t_{2}\right)$ can be obtained from Equation (9), $△E^{m}\left(t_{0},t_{1}\right)$ and $△E^{m,l}\left(t_{0},t_{1},t_{2}\right)$ can be derived from Equation (1). And $△E_{blind}\left(t_{0}\right)$ can be obtained from Equation (2). If $△E_{1,2}^{m,l}$$-\sum_{n}△E_{0,1,2}^{n,m,l}\neq 0$, macrorealism can not be satisfied. The meanings of terms in Equations (6) and (8) are similar to those corresponding terms in Equation (7).

When the CNSIT conditions are satisfied, the energy change of the system in the whole process does not depend on whether any prior measurement has been performed or not. In fact, the CNSIT conditions are based on comparing the conditional energy change of the system in the whole process, in the cases where a previous measurement has or has not been performed at some time. And similar to the NSIT conditions, the violation of one of the CNSIT conditions means the violation of NIM, which implies the violation of macrorealism. That is to say, when one of the CNSIT conditions is violated, macrorealism is violated.

### 2.4. ANSIT Conditions

It is noted that multiplying the change of conditional energy by its measurement probability and then summing all results, we obtain the corresponding change of average energy. Next, analogous to the CNSIT conditions, the two-time and three-time ANSIT conditions can be given as

(10) $$△{\bar{E}}_{(i)j}:△{\bar{E}}_{j}-△{\bar{E}}_{i,j}=0,$$

(11) $$△{\bar{E}}_{(0)1,2}:△{\bar{E}}_{1,2}-△{\bar{E}}_{0,1,2}=0,$$

(12) $$△{\bar{E}}_{0(1),2}:△{\bar{E}}_{0,2}-△{\bar{E}}_{0,1,2}=0.$$

Similarly, we also take $△{\bar{E}}_{(0)1,2}$ in Equation (11) as an example to explain the ANSIT conditions (see Appendix A). $△{\bar{E}}_{1,2}$ and $△{\bar{E}}_{0,1,2}$ in $△{\bar{E}}_{(0)1,2}$ (Equation (11)) are the average energy change of the system in the whole process, when a single observable is measured at two time steps and three time steps, respectively. And both $△{\bar{E}}_{(i)j}$ and $△{\bar{E}}_{0(1),2}$ of Equations (10) and (12) are similar to $△{\bar{E}}_{(0)1,2}$.

Similar to the CNSIT conditions, the ANSIT conditions compare the average energy change of the whole process in the cases that a measurement previously has or has not been performed at some time. And when the ANSIT conditions are satisfied, the average energy change of the system in the whole process is not dependent on whether any prior measurement has been performed. The violation of one of equalities in Equations (10) to (12) means that the assumption of NIM is not satisfied. And in this situation, macrorealism is violated.

## 3. A Pure Qubit

Firstly, we consider coherent dynamics of a qubit described by a Hamiltonian $H=\frac{1}{2}\omega \sigma_{z}$ (ℏ=1), where $\sigma_{z}$ and ω are the Pauli operator and the energy gap of the qubit, respectively. We suppose that the measurement of a dichotomic observable is equivalent to a measurement of the Bloch sphere component along a direction of θ and ϕ, i.e., $Q\left(\theta ,\phi \right)=Q^{+}\left(\theta ,\phi \right)-Q^{-}\left(\theta ,\phi \right)$. Here,

(13) $$\begin{array}{ccc} Q^{+}\left(\theta ,\phi \right) & = & |n^{+}\left(\theta ,\phi \right)\rangle \langle n^{+}\left(\theta ,\phi \right)|, \end{array}$$

(14) $$\begin{array}{ccc} Q^{-}\left(\theta ,\phi \right) & = & |n^{-}\left(\theta ,\phi \right)\rangle \langle n^{-}\left(\theta ,\phi \right)|, \end{array}$$

where $|n^{+}\left(\theta ,\phi \right)\rangle$ $=\cos\frac{\theta }{2}|0\rangle +e^{i\phi }\sin\frac{\theta }{2}|1\rangle$ and $|n^{-}\left(\theta ,\phi \right)\rangle =\sin\frac{\theta }{2}|0\rangle -e^{i\phi }\cos\frac{\theta }{2}|1\rangle$ (θ∈[0,π), ϕ∈[0,2π)), with |0〉 and |1〉 being the eigenstates of the Pauli operator $\sigma_{z}$. Therefore, these probabilities $P(Q^{m}\left(t_{j}\right))$, $P(Q^{n}\left(t_{i}\right),Q^{m}\left(t_{j}\right))$ and $P(Q^{n}\left(t_{0}\right),Q^{m}\left(t_{1}\right),Q^{l}\left(t_{2}\right))$ in Equations (3) to (5) can be written as

(15) $$P\left(Q^{m}\left(t_{j}\right)\right)=\mathrm{Tr}\left[Q^{m}\left(t_{j}\right)U\left(t_{j},0\right)\rho \left(0\right)U^{†}\left(t_{j},0\right){Q^{m}}^{†}\left(t_{j}\right)\right],$$

(16) $$P\left(Q^{n}\left(t_{i}\right),Q^{m}\left(t_{j}\right)\right)=\mathrm{Tr}\left[Q^{m}\left(t_{j}\right)U\left(t_{j},t_{i}\right)Q^{n}\left(t_{i}\right)U\left(t_{i},0\right)\rho \left(0\right)U^{†}\left(t_{i},0\right)Q^{n†}\left(t_{i}\right)U^{†}\left(t_{j},t_{i}\right)Q^{m†}\left(t_{j}\right)\right],$$

(17) $$P\left(Q^{n}\left(t_{0}\right),Q^{m}\left(t_{1}\right),Q^{l}\left(t_{2}\right)\right)=\mathrm{Tr}[Q^{l}\left(t_{2}\right)U\left(t_{2},t_{1}\right)Q^{m}\left(t_{1}\right)U\left(t_{1},t_{0}\right)Q^{n}\left(t_{0}\right)U\left(t_{0},0\right)\rho \left(0\right)\;\times U^{†}\left(t_{0},0\right)Q^{n†}\left(t_{0}\right)U^{†}(t_{1},t_{0})Q^{m†}\left(t_{1}\right)U^{†}\left(t_{2},t_{1}\right)Q^{l†}\left(t_{2}\right)],$$

where n,m,l=±1, ρ(0) is the initial state, and $U\left(t,0\right)=\exp\left[-\frac{i}{2}\omega \sigma_{z}t\right]$ is the unitary operator. Without loss of generality, in the subsequent analysis, we suppose $t_{0}=0$ and $t_{2}-t_{1}=t_{1}-t_{0}=\left(1/2\right)\left(t_{2}-t_{0}\right)=\tau$.

### NSIT Conditions, CNSIT Conditions and ANSIT Conditions for Initial Mixed and Pure States

Next, we take the initial state as a mixed state, such that

(18) $$\rho \left(0\right)=\frac{1-\delta }{2}\left|0\rangle \langle 0\right|+\frac{1+\delta }{2}|1\rangle \langle 1|,$$

where 0≤δ≤1. From Equations (3) to (5), and (13) to (18), we obtain the NSIT conditions in Equations (3) to (5) (see Appendix B). Next, from Equations (1), (2), (6) to (9), (13), (14) and (18), we obtain two-time and three-time CNSIT conditions in Equations (6) to (8) (see Appendix C). The ANSIT conditions in Equations (10) to (12) can be obtained from Equations (2), (9) to (14), and (18), (see Appendix D). Then, we list non-violation conditions of the NSIT conditions, the CNSIT conditions and the ANSIT conditions in Table 1. It is noted that when $\tau =\frac{2\pi }{\omega }$, the NSIT conditions can not be violated. And the evolution of this system is unitary, thus when $\tau =\frac{2k\pi }{\omega }$(k=1,2,3…), the NSIT conditions are satisfied. For simplicity, we suppose $\tau \in [0,\frac{2\pi }{\omega }]$ in the following.

It is noted that when measurement operators $Q^{+}$ and $Q^{-}$ are performed on arbitrary state, the post-measurement states are $|n^{+}\left(\theta ,\phi \right)\rangle \langle n^{+}\left(\theta ,\phi \right)|$ and $|n^{-}\left(\theta ,\phi \right)\rangle \langle n^{-}\left(\theta ,\phi \right)|$, i.e., $Q^{+}$ and $Q^{-}$ (Equations (13) and (14)), respectively. Comparing the non-violation conditions of the NSIT conditions, the CNSIT conditions and the ANSIT conditions, we find that the ANSIT conditions can not be violated for a wider parameter regime than the NSIT conditions and the CNSIT conditions. And it is interesting to find that the non-violation conditions of the NSIT conditions and the CNSIT conditions do not contain each other, which can be seen from Table 1. This can be explained as following:

1. *When* $\tau =\frac{2\pi }{\omega }$*, the NSIT conditions are satisfied, while the CNSIT conditions are not.*

For the NSIT conditions, we take $P\left(Q^{+}\left(t_{1}\right)\right)-\sum_{\pm }P\left(Q^{\pm }\left(t_{0}\right),Q^{+}\left(t_{1}\right)\right)$ (Equation (3)) as an example to explain it. It is noted that when $\tau =\frac{2\pi }{\omega }$, the unitary operator is the identity operator, i.e., the unitary operator has no effect on the state. Therefore, $P\left(Q^{+}\left(t_{1}\right)\right)=P\left(Q^{+}\left(t_{0}\right),Q^{+}\left(t_{1}\right)\right)$, and $P(Q^{-}\left(t_{0}\right),Q^{+}\left(t_{1}\right))=0$, so $P\left(Q^{+}\left(t_{1}\right)\right)-\sum_{\pm }P\left(Q^{\pm }\left(t_{0}\right),Q^{+}\left(t_{1}\right)\right)=0$. And other NSIT conditions are similar to it. Then, for the CNSIT conditions, we take $△E_{1}^{+}-\sum_{\pm }△E_{0,1}^{\pm ,+}$ in Equation (6) as an example. Because the unitary operator has no effect on the state, the energy change of evolution is zero. And the energy change before and after the second measurement at $t=t_{1}$ is zero, thus, $\sum_{\pm }△E_{0,1}^{\pm ,+}$ is decided by the energy change before and after the blind measurement at $t=t_{0}$, i.e., $\sum_{\pm }△E_{0,1}^{\pm ,+}=△E_{blind}\left(t_{0}\right)$. While for $△E_{1}^{+}$ (Equation (6)), we find that $△E_{1}^{+}\neq △E_{blind}\left(t_{0}\right)$, i.e., $△E_{1}^{+}\neq \sum_{\pm }△E_{0,1}^{\pm ,+}$, thus, when $\tau =\frac{2\pi }{\omega }$, $△E_{(0)1}^{(\pm )+}$(Equation (6)) is not satisfied, and other CNSIT conditions are similar to it.

2. *When* $\theta =\frac{\pi }{2}$*, the CNSIT conditions can not be violated, while the NSIT conditions are violated (*$\tau \neq \frac{\pi }{\omega }$ *and* $\tau \neq \frac{2\pi }{\omega }$*).*

For the CNSIT conditions, we also take $△E_{1}^{+}-\sum_{\pm }△E_{0,1}^{\pm ,+}$ (Equation (6)) as an example. It is noted that when $\theta =\frac{\pi }{2}$, the measurement operator is in the x-y plane of the Bloch sphere, and both diagonal elements of post-measurement states by blind measurement ($\theta =\frac{\pi }{2}$) for arbitrary state are $\frac{1}{2}$. And then the diagonal elements of post-measurement states can not be affected by the subsequent measurements. Therefore, the energy change before and after the second measurement at $t=t_{1}$ for $\sum_{\pm }△E_{0,1}^{\pm ,+}$ is zero, and $\sum_{\pm }△E_{0,1}^{\pm ,+}$ in Equation (6) equals to the energy change before and after the blind measurement at $t=t_{0}$. Similarly, $\sum_{\pm }△E_{0,1}^{\pm ,+}=△E_{blind}\left(t_{0}\right)$. Then, for $△E_{1}^{+}$ (Equation (6)), we find that $△E_{1}^{+}=△E_{blind}\left(t_{0}\right)=\sum_{\pm }△E_{0,1}^{\pm ,+}$, so $△E_{1}^{+}-\sum_{\pm }△E_{0,1}^{\pm ,+}=0$. Therefore, when $\theta =\frac{\pi }{2}$, $△E_{(0)1}^{(\pm )+}$ in (Equation (6)) is satisfied, and other CNSIT conditions are similar to it. While for the NSIT conditions, when $\theta =\frac{\pi }{2}$, except for $\tau =\frac{\pi }{\omega }$ (the unitary operator is the Pauli operator $\sigma_{z}$) and $\tau =\frac{2\pi }{\omega }$, they are violated. Then, we take $P\left(Q^{+}\left(t_{0}\right),Q^{+}\left(t_{2}\right)\right)-\sum_{\pm }P\left(Q^{+}\left(t_{0}\right),Q^{\pm }\left(t_{1}\right),Q^{+}\left(t_{2}\right)\right)$ in Equation (5) as an example. When $\theta =\frac{\pi }{2}$, $P\left(Q^{+}\left(t_{0}\right),Q^{+}\left(t_{2}\right)\right)-\sum_{\pm }P\left(Q^{+}\left(t_{0}\right),Q^{\pm }\left(t_{1}\right),Q^{+}\left(t_{2}\right)\right)=-\frac{1}{4}\sin^{2}\tau \omega$, so for $\theta =\frac{\pi }{2}$, only when $\tau =\frac{\pi }{\omega }$ ($\frac{2\pi }{\omega }$), $P\left(Q^{+}\left(t_{0}\right),Q^{+}\left(t_{2}\right)\right)-\sum_{\pm }P\left(Q^{+}\left(t_{0}\right),Q^{\pm }\left(t_{1}\right),Q^{+}\left(t_{2}\right)\right)=0$. And other NSIT conditions are similar to it. The reason of this phenomenon is that the NSIT conditions consider the probability of measurement, while the CNSIT conditions consider the change of energy.

Now, we consider a pure state

(19) |φ〉=cosψ|0〉+sinψ|1〉,

where ψ∈[0,π). Notably, for the initial pure state, it is too cumbersome to list every term of the NSIT conditions, the CNSIT conditions and the ANSIT conditions (Equations (3) to (5), (6) to (8), and (10) to (12)). Therefore, for the sake of simplicity, we only list non-violation conditions of the NSIT conditions, the CNSIT conditions and the ANSIT conditions in Table 1.

In a word, we find that the non-violation conditions of the NSIT conditions for both initial mixed and pure states are the same. While for the non-violation conditions of the CNSIT conditions and the ANSIT conditions, they are both dependent on the initial state. And the CNSIT conditions for the mixed state can not be violated for a wider parameter regime than that of the pure state. In addition, we also find that whether the initial state is the mixed state or the pure state, the ANSIT conditions can not be violated for a wider parameter regime than the NSIT conditions and the CNSIT conditions. And the non-violation conditions of the NSIT conditions and the CNSIT conditions do not contain each other. In other words, a suitable conjunction of the NSIT conditions and the CNSIT conditions may be better for testing macrorealism (MR). Therefore, for both initial mixed and pure states, we summarize the main result of this section as follows:

(20) $$NSIT\;\mathrm{or}\;CNSIT\Rightarrow ANSIT,NSIT\cap CNSIT\overset{better}{\Rightarrow }MR.$$

**Figure 2:**
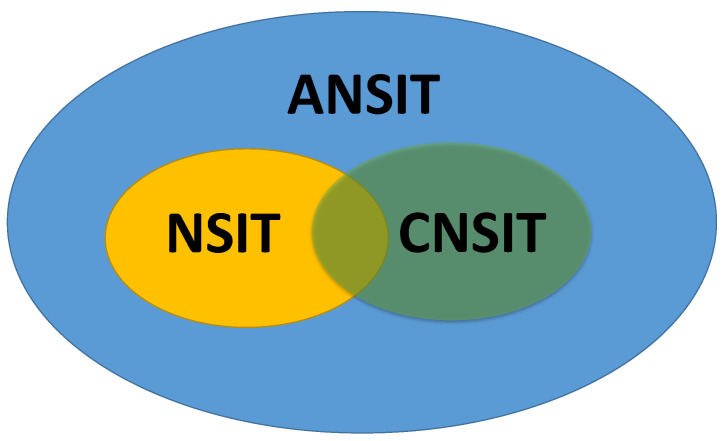
Schematic diagram for the relationship among the NSIT conditions, the CNSIT conditions and the ANSIT conditions in the case of the pure qubit and the dephasing qubit, respectively.

And the relationship among the NSIT conditions, the CNSIT conditions and the ANSIT conditions can be seen from Figure 2. Recently, Smirne et al. [30] gave a definite criteria to determine when and to what extent quantum coherence is equivalent to non-classicality, i.e., a notion of coherence-generating-and-detecting (CGD) dynamics. CGD means that the evolution does generate coherence, and can also turn such coherence into the populations which is measured at a later time; otherwise, the evolution is denoted as NCGD. Then, we find that for the initial mixed state, when the NSIT conditions, the CNSIT conditions, and the ANSIT conditions are not violated, the evolution of the system is NCGD, which means that the evolution does not generate coherence, and can not also turn such coherence into the populations for subsequent measurements; while for the initial pure state, when the NSIT conditions, the CNSIT conditions, and the ANSIT conditions are not violated, the evolution of the system might be NCGD or CGD, i.e., the non-violations of these three kinds of conditions and NCGD do not contain each other.

## 4. A Driven Qubit

Next, we consider a resonant, periodic driven two-level system and its time-dependent Hamiltonian is described as [31,32]

(21) $$H\left(t\right)=\frac{1}{2}\omega \sigma_{z}+\frac{g}{2}\left[\sigma_{x}\cos\omega t+\sigma_{y}\sin\omega t\right].$$

Here, $\sigma_{x}$ and $\sigma_{y}$ are both Pauli operators, ω>0 is the energy gap of the qubit as before and also the driving frequency of the two-level system, and g∈[0,ω] is the driving intensity quantifying the coupling to the external field. The system evolves under the unitary operator $U\left(t,0\right)=\overset{\leftarrow }{T}\exp\left[-i\int_{0}^{t}d\tau H\left(\tau \right)\right]$, where $\overset{\leftarrow }{T}$ is a time-ordering operator. The unitary operator can be expressed as [31,32]

(22) $$U\left(t,0\right)=\left(\begin{array}{cc} e^{-\frac{1}{2}it\omega }\cos\left(\frac{gt}{2}\right) & -ie^{-\frac{1}{2}it\omega }\sin\left(\frac{gt}{2}\right)\\ -ie^{\frac{it\omega }{2}}\sin\left(\frac{gt}{2}\right) & e^{\frac{it\omega }{2}}\cos\left(\frac{gt}{2}\right) \end{array}\right),$$

which satisfies $U^{†}\left(t,0\right)U\left(t,0\right)=I$ and $U\left(t_{j},t_{i}\right)U\left(t_{i},0\right)=U\left(t_{j},0\right)$ with i<j. It is noted that the NSIT conditions, the CNSIT conditions and the ANSIT conditions for the driven two-level system are very complicated, we thus suppose g=0.1ω, g=0.5ω and g=ω to study the violations of them.

**Figure 3:**
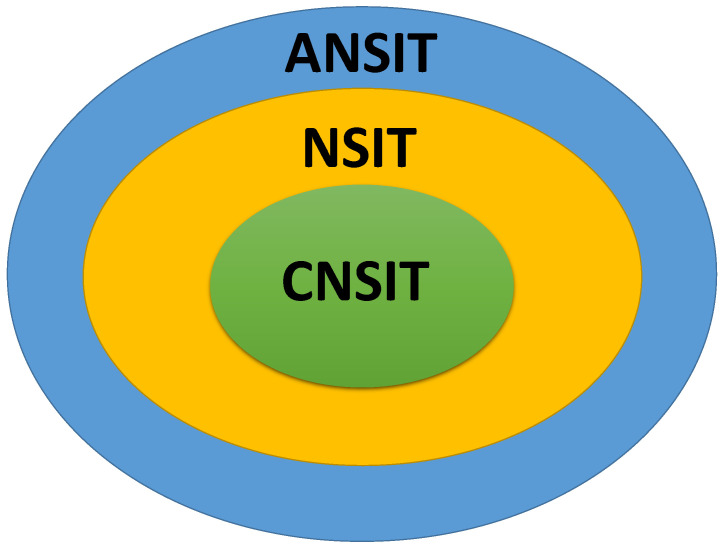
Schematic diagram for the relationship among the NSIT conditions, the CNSIT conditions and the ANSIT conditions in the case of the driven qubit and the dissipative qubit, respectively.

In short, we find that similar to the qubit without drive, the non-violation conditions of the NSIT conditions for the initial mixed state are the same as that of the pure state; and the non-violation conditions of the CNSIT conditions and the ANSIT conditions are both dependent on the initial state. Furthermore, we also find that the CNSIT conditions can not be violated for a narrower range of parameters than the NSIT conditions, and the NSIT conditions can not be violated for a narrower parameter regime than the ANSIT conditions, for both initial mixed and pure states. The above phenomenon is summarized as follows:

(23) CNSIT⇒NSIT⇒ANSIT,

which can be seen from Figure 3.

## 5. A Qubit Interacting with Environment

As we know, quantum systems inevitably suffer from unwanted interactions with environment, so in this section, we study the effects of environment on violations of the NSIT conditions, the CNSIT conditions and the ANSIT conditions. The time evolution of open system is different from the closed system discussed in the previous sections, which in general can not be described by a unitary time evolution. The dynamics of the system can be represented by an appropriate equation of motion for its density matrix, i.e., a quantum master equation. In this case, the evolution of the system is provided most generally by the Lindblad form master equation, which can be written as

(24) $$\frac{d\rho }{dt}=-i\left[H,\rho \right]+\sum_{k}\left[2L_{k}\rho L_{k}^{†}-L_{k}^{†}L_{k}\rho -\rho L_{k}^{†}L_{k}\right],$$

where the Hamiltonian $H=\frac{1}{2}\omega \sigma_{z}$, as in the preceding section, represents the coherent part of the dynamics, and $L_{k}$ is the Lindblad operator describing the coupling of the system to its environment.

### 5.1. A Dissipative Qubit

We consider the first case, that the Lindblad operator is $L_{k}=\sqrt{\gamma }\sigma_{-}$, where γ≥0 is the rate of spontaneous emission, and $\sigma_{-}=\left|1\rangle \langle 0\right|$ is the atomic lowering operator. The master equation describing this process can be rewritten as

(25) $$\frac{d\rho }{dt}=-i\left[H,\rho \right]+\gamma \left[2\sigma_{-}\rho \sigma_{-}^{†}-\sigma_{-}^{†}\sigma_{-}\rho -\rho \sigma_{-}^{†}\sigma_{-}\right].$$

From Equations (3) to (5), (13) to (18), and (25), we obtain all the NSIT conditions for the initial mixed state. For simplicity, we give two examples for the NSIT conditions in the following. According to ${\mathrm{NSIT}}_{(0)1}^{(\pm )+}$ (Equation (3)) and ${\mathrm{NSIT}}_{0(1)2}^{+(\pm )+}$ (Equation (5)), we can respectively obtain $P\left(Q^{+}\left(t_{1}\right)\right)-\sum_{\pm }P\left(Q^{\pm }\left(t_{0}\right),Q^{+}\left(t_{1}\right)\right)$ and $P\left(Q^{+}\left(t_{0}\right),Q^{+}\left(t_{2}\right)\right)-\sum_{\pm }P\left(Q^{+}\left(t_{0}\right),Q^{\pm }\left(t_{1}\right),Q^{+}\left(t_{2}\right)\right)$ as

(26) $$P\left(Q^{+}\left(t_{1}\right)\right)-\sum_{\pm }P\left(Q^{\pm }\left(t_{0}\right),Q^{+}\left(t_{1}\right)\right)=\frac{1}{8}\delta \sin\theta \sin2\theta e^{-\gamma \tau }\left(-2e^{-\gamma \tau }+e^{-i\tau \omega }+e^{i\tau \omega }\right),$$

$$P\left(Q^{+}\left(t_{0}\right),Q^{+}\left(t_{2}\right)\right)-\sum_{\pm }P\left(Q^{+}\left(t_{0}\right),Q^{\pm }\left(t_{1}\right),Q^{+}\left(t_{2}\right)\right)$$

$$=-\frac{1}{32}\left(\delta \cos\theta -1\right)e^{-4\gamma \tau -2i\tau \omega }\sin^{2}\theta [-4e^{\tau (\gamma +i\omega )}-2e^{2\tau (\gamma +i\omega )}+3e^{2\tau (\gamma +2i\omega )}+3e^{2\gamma \tau }-4e^{\tau (\gamma +3i\omega )}$$

(27) $$\begin{array}{c} +4e^{2i\tau \omega }-\cos2\theta {\left(e^{\tau (\gamma +2i\omega )}+e^{\gamma \tau }-2e^{i\tau \omega }\right)}^{2}+4\cos\theta \left(e^{2\gamma \tau }-1\right)e^{i\tau \omega }\left(e^{\tau (\gamma +2i\omega )}+e^{\gamma \tau }-2e^{i\tau \omega }\right)]. \end{array}$$

Other NSIT conditions are similar to Equations (26) and (27), respectively. It can be found from Equation (26) that when θ=0 and $\theta =\frac{\pi }{2}$, ${\mathrm{NSIT}}_{(0)1}^{(\pm )+}$ is satisfied. For ${\mathrm{NSIT}}_{0(1)2}^{+(\pm )+}$ (Equation (27)), when θ=0, it is satisfied. However, when $\theta =\frac{\pi }{2}$, $P\left(Q^{+}\left(t_{0}\right),Q^{+}\left(t_{2}\right)\right)-\sum_{\pm }P\left(Q^{+}\left(t_{0}\right),Q^{\pm }\left(t_{1}\right),Q^{+}\left(t_{2}\right)\right)=-\frac{1}{4}e^{-2\gamma \tau }\sin^{2}\left(\tau \omega \right)$, i.e., ${\mathrm{NSIT}}_{0(1)2}^{+(\pm )+}$ is not satisfied. And it can not be violated if $\theta =\frac{\pi }{2}$ and $\tau =\frac{\pi }{\omega }$ ($\frac{2\pi }{\omega }$). Then, considering other NSIT conditions, we find that all the NSIT conditions can not be violated when one of the conditions is satisfied: (1) θ=0; (2) $\theta =\frac{\pi }{2}$ and $\tau =\frac{\pi }{\omega }$ ($\frac{2\pi }{\omega }$). Furthermore, we find that for all the NSIT conditions, there is a factor $e^{-\gamma \tau }$, which can be seen from Equations (26) and (27). That is to say, the degree of violation of the NSIT conditions is decreased by dissipation with time interval between measurements τ increasing. And when τ→∞, all the NSIT conditions are satisfied, which also can be seen from Equations (26) and (27). It has been mentioned in Section 3 that when $\tau =\frac{2k\pi }{\omega }$(k=1,2,3…), the NSIT conditions are satisfied for the pure qubit (see Table 1). While in the presence of dissipation, the evolution of the system is not unitary, thus the NSIT conditions are not satisfied when $\tau =\frac{2k\pi }{\omega }$(k=1,2,3…). In addition, we find that similar to the NSIT conditions, the degree of violations of the CNSIT conditions and the ANSIT conditions is decreased by dissipation with τ increasing. And when τ→∞, the CNSIT conditions and the ANSIT conditions can not be violated.

Then, we obtain the non-violation conditions of the NSIT conditions, the CNSIT conditions and the ANSIT conditions for the initial pure state, which are the same as that of the initial mixed state and are listed in Table 1. In addition, we find that similar to the driven qubit, the non-violation of the CNSIT conditions implies the non-violation of the NSIT conditions, which in turn implies the non-violation of the ANSIT conditions, i.e.,

(28) CNSIT⇒NSIT⇒ANSIT,

which also can be seen from Figure 3. For driven and dissipative qubits, the energy changes due to coupling to the external field and the environment, which might be the reason why the relationship among the NSIT conditions, the CNSIT conditions and the ANSIT conditions for the dissipative qubit is similar to that of the driven qubit.

### 5.2. A Dephasing Qubit

Let us consider the second case, i.e., the Lindblad operator is $L_{k}=\sqrt{\gamma }\sigma_{z}$. And the Lindblad master equation which describes this process, can be rewritten as

(29) $$\frac{d\rho }{dt}=-i\left[H,\rho \right]+\gamma \left[2\sigma_{z}\rho \sigma_{z}^{†}-\sigma_{z}^{†}\sigma_{z}\rho -\rho \sigma_{z}^{†}\sigma_{z}\right].$$

Similarly, we list the non-violation conditions of the NSIT conditions, the CNSIT conditions and the ANSIT conditions for both initial mixed and pure states in Table 1. We find that the non-violation conditions of the CNSIT conditions and the ANSIT conditions depend on the initial state, which is different from dissipation. The reason is that dephasing only removes the non-diagonal elements of the initial state, while its diagonal elements do not change. Then, for both initial mixed and pure states, we find that the relationship among the NSIT conditions, the CNSIT conditions and the ANSIT conditions is similar to that of coherent dynamics (see Figure 2), i.e.,

(30) $$NSIT\;\mathrm{or}\;CNSIT\Rightarrow ANSIT,NSIT\cap CNSIT\overset{better}{\Rightarrow }MR.$$

The reason of above phenomenon might be that for dephasing, the energy of the system conserves, which is similar to the pure qubit.

In addition, we also find that similar to the dissipative qubit, when $\tau =\frac{2k\pi }{\omega }$(k=1,2,3…), the NSIT conditions for dephasing are violated; with τ increasing, the degree of violations of the NSIT conditions, the CNSIT conditions and the ANSIT conditions is also decreased by dephasing; and when τ→∞, the NSIT conditions, the CNSIT conditions and the ANSIT conditions are also satisfied. Furthermore, comparing the dephasing qubit with the dissipative qubit, we find that when the NSIT conditions, the CNSIT conditions and the ANSIT conditions for dissipation are not violated, these three kinds of conditions for dephasing must not be violated, respectively, for both initial mixed and pure states.

## 6. Conclusions

As we all know, the Leggett–Garg inequality can not provide the necessary and sufficient conditions for macrorealism, and the NSIT conditions are also necessary conditions for macrorealism. The Leggett–Garg inequality puts limits on temporal correlation functions of pairs of consecutive measurements on the same quantum system fulfilled macrorealism, and the NSIT conditions say that a measurement does not change the outcome statistics of a later measurement. In this paper, we probe the boundaries of classical and quantum physics from a new perspective. We use energy change to test macrorealism, and analogous to the NSIT conditions, we give a set of equalities for the change of energy. In this paper, we consider a qubit in four scenarios: with drive, without drive, in the presence of dissipation, and in the presence dephasing to investigate the violations of the NSIT conditions, the CNSIT conditions and the ANSIT conditions. From this simple model, we find that the non-violation conditions of the CNSIT conditions and the ANSIT conditions both depend on the initial state (except for dissipation), while the non-violation conditions of the NSIT conditions for the initial mixed state is the same as that of the initial pure state. For coherent dynamics with and without drive, the dynamics under dissipation and dephasing, the ANSIT conditions are satisfied for a wider parameter regime than that of the NSIT conditions and the CNSIT conditions, whether the initial state is mixed state or pure state. While for the non-violations of the NSIT conditions and the CNSIT conditions, the four scenarios fall into two groups: one is that the non-violation conditions of the NSIT conditions and CNSIT conditions do not contain each other for the pure and dephasing qubits; the other is that the NSIT conditions can not be violated for a wider parameter regime than that of the CNSIT conditions for the driven and dissipative qubits. Therefore, we find that the NSIT and CNSIT conditions are not equivalent, i.e., the NSIT conditions and the CNSIT conditions complement each other, and the conjunction of them can be better to test macrorealism. The above phenomena might because that for the former the energy of the system is conserved, while for the latter it is not. We guess that this might be a general result, but needs further study. In the future, we will gain some insight into it, and hope to find a more general proof of the logical connection among these three kinds of conditions. Comparing dissipative and dephasing qubits, we find that the non-violation conditions of the CNSIT conditions and the ANSIT conditions for dissipation do not depend on the initial state, while for dephasing, non-violation conditions of these kinds of conditions depend on the initial state. This is because dephasing only removes the non-diagonal elements of the initial state, but its diagonal elements do not change. Furthermore, we find that when the non-violations of the NSIT conditions, the CNSIT conditions and the ANSIT conditions for dissipation, are satisfied, these three kinds of conditions for dephasing must be satisfied, respectively. In addition, dissipation and dephasing both decrease the degree of violations of the NSIT conditions, the CNSIT conditions and the ANSIT conditions, with time interval between measurements τ increasing; and when τ→∞, the NSIT conditions, the CNSIT conditions and the ANSIT conditions are satisfied.

In quantum information and computation, coherence and entanglement are the essential ingredients, which both arise from quantum superposition principle. And quantum coherence is a more basic trait in quantum mechanics, which allows us to distinguish between classical and quantum phenomena. The Bell inequality is related to quantum correlations, while the Leggett–Garg inequality is related to coherence. Quantum coherence is a more basic resource in quantum-information processing. The Leggett–Garg inequality, the NSIT conditions, the CNSIT conditions and the ANSIT conditions are all used to observe when physical systems stop to behave quantumly and begin to behave classically. In other words, they can help us to know in a quantum dynamical process when quantum resource can be used and when it is not.

## Figures and Tables

**Table 1:** The non-violation conditions of the NSIT conditions, the CNSIT conditions and the ANSIT conditions for coherent dynamics, dynamics under dissipation, and dynamics under dephasing, for the initial mixed and pure states, respectively.

**coherent dynamics**	**mixed state**	**pure state**
NSIT conditions	1. θ=0; 2. $\tau =\frac{2\pi }{\omega };$ 3. $\theta =\frac{\pi }{2}$ and $\tau =\frac{\pi }{\omega };$	
CNSIT conditions		1. θ=0;
	1. θ=0;	2. $\theta =\frac{\pi }{2}$ and ψ=0;
	2. $\theta =\frac{\pi }{2};$	3. $\theta =\frac{\pi }{2}$ and $\psi =\frac{\pi }{4};$
		4. $\theta =\frac{\pi }{2}$ and $\psi =\frac{\pi }{2};$
		5. $\theta =\frac{\pi }{2}$ and $\psi =\frac{3\pi }{4};$
ANSIT conditions		1. θ=0;
	1. θ=0;	2. $\tau =\frac{2\pi }{\omega };$
	2. $\tau =\frac{2\pi }{\omega };$	3. $\theta =\frac{\pi }{2}$;
	3. $\theta =\frac{\pi }{2}$;	4. $\tau =\frac{\pi }{\omega }$, $\phi =\frac{\pi }{2}$ and $\psi =\frac{\pi }{4}$;
	4. δ=0;	5.$\tau =\frac{\pi }{\omega }$, $\phi =\frac{\pi }{2}$ and $\psi =\frac{3\pi }{4}$;
		6. $\tau =\frac{\pi }{\omega }$, $\phi =\frac{3\pi }{2}$ and $\psi =\frac{\pi }{4}$;
		7. $\tau =\frac{\pi }{\omega }$, $\phi =\frac{3\pi }{2}$ and $\psi =\frac{3\pi }{4}$;
**dynamics under dissipation**		
NSIT conditions	1. θ=0; 2. $\theta =\frac{\pi }{2}$ and $\tau =\frac{\pi }{\omega };$ 3. $\theta =\frac{\pi }{2}$ and $\tau =\frac{2\pi }{\omega };$	
CNSIT conditions	θ=0;	
ANSIT conditions	1. θ=0; 2. $\theta =\frac{\pi }{2}$	
**dynamics under dephasing**		
NSIT conditions	1. θ=0; 2. $\theta =\frac{\pi }{2}$ and $\tau =\frac{\pi }{\omega };$ 3. $\theta =\frac{\pi }{2}$ and $\tau =\frac{2\pi }{\omega };$	
CNSIT conditions		1. θ=0;
	1. θ=0;	2. $\theta =\frac{\pi }{2}$ and ψ=0;
	2. $\theta =\frac{\pi }{2}$	3. $\theta =\frac{\pi }{2}$ and $\psi =\frac{\pi }{4};$
		4. $\theta =\frac{\pi }{2}$ and $\psi =\frac{\pi }{2};$
		5. $\theta =\frac{\pi }{2}$ and $\psi =\frac{3\pi }{4};$
ANSIT conditions		1. θ=0;
		2. $\theta =\frac{\pi }{2}$;
		3. $\tau =\frac{\pi }{\omega }$, $\phi =\frac{\pi }{2}$ and $\psi =\frac{\pi }{4}$;
	1. θ=0;	4. $\tau =\frac{\pi }{\omega }$, $\phi =\frac{\pi }{2}$ and $\psi =\frac{3\pi }{4}$;
	2. $\theta =\frac{\pi }{2}$;	5. $\tau =\frac{\pi }{\omega }$, $\phi =\frac{3\pi }{2}$ and $\psi =\frac{\pi }{4}$;
	3. δ=0;	6. $\tau =\frac{\pi }{\omega }$, $\phi =\frac{3\pi }{2}$ and $\psi =\frac{3\pi }{4}$;
		7. $\tau =\frac{2\pi }{\omega }$, $\phi =\frac{\pi }{2}$ and $\psi =\frac{\pi }{4}$;
		8. $\tau =\frac{2\pi }{\omega }$, $\phi =\frac{\pi }{2}$ and $\psi =\frac{3\pi }{4}$;
		9. $\tau =\frac{2\pi }{\omega }$, $\phi =\frac{3\pi }{2}$ and $\psi =\frac{\pi }{4}$;
		10. $\tau =\frac{2\pi }{\omega }$, $\phi =\frac{3\pi }{2}$ and $\psi =\frac{3\pi }{4}$;

## Abbreviations

The following abbreviations are used in this manuscript:

NSIT	no-signaling-in-time
CNSIT	no-signaling-in-time conditions for conditional energy
ANSIT	no-signaling-in-time conditions for average energy
MRps	Macrorealism per se
NIM	Non-invasive measurability
NS	no-signaling
MR	macrorealism
CGD	coherence-generating-and-detecting

## Appendix A. ANSIT Conditions

The average energy change $△{\bar{E}}_{1,2}$ of Equation (11) is consist of:

I. the change of energy $△{\bar{E}}_{evo}\left(0\to t_{1}\right)$ during the evolution from t=0 to $t=t_{1}$;

II. the change of average energy $△\bar{E}\left(t_{1}\right)$, which is the difference of average energy before and after blind measurements at $t=t_{1}$;

III. the change of energy $△{\bar{E}}_{evo}\left(t_{1}\to t_{2}\right)$ during the evolution from $t=t_{1}$ to $t=t_{2}$;

IV. the change of average energy $△\bar{E}\left(t_{2}\right)$ before and after blind measurement at $t=t_{2}$.

Therefore, $△{\bar{E}}_{1,2}=△{\bar{E}}_{evo}\left(0\to t_{1}\right)+△\bar{E}\left(t_{1}\right)+△{\bar{E}}_{evo}\left(t_{1}\to t_{2}\right)+△\bar{E}\left(t_{2}\right)$, where $△{\bar{E}}_{evo}\left(0\to t_{1}\right)$ and $△{\bar{E}}_{evo}\left(t_{1}\to t_{2}\right)$ can be obtained from Equation (9), $△\bar{E}\left(t_{1}\right)$ and $△\bar{E}\left(t_{2}\right)$ can be derived from Equation (2).

Next, the average energy change $△{\bar{E}}_{0,1,2}$ of Equation (11) includes:

I. the energy change $△{\bar{E}}_{evo}\left(0\to t_{0}\right)$ during the evolution from t=0 to $t=t_{0}$;

II. the average energy change $△\bar{E}\left(t_{0}\right)$ before and after blind measurement at $t=t_{0}$;

III. the energy change $△{\bar{E}}_{evo}\left(t_{0}\to t_{1}\right)$ during the evolution from $t=t_{0}$ to $t=t_{1}$;

IV. the average energy change before and after blind measurement at $t=t_{1}$, $\bar{E}\left(t_{1}\right)$;

V. the energy change $△{\bar{E}}_{evo}\left(t_{1}\to t_{2}\right)$ during the evolution from $t=t_{1}$ to $t=t_{2}$;

VI. the average energy change $△\bar{E}\left(t_{2}\right)$ before and after blind measurement at $t=t_{2}$.

Therefore, $△{\bar{E}}_{0,1,2}=△{\bar{E}}_{evo}\left(0\to t_{0}\right)+△\bar{E}\left(t_{0}\right)+△{\bar{E}}_{evo}\left(t_{0}\to t_{1}\right)+△\bar{E}\left(t_{1}\right)+△{\bar{E}}_{evo}\left(t_{1}\to t_{2}\right)+△\bar{E}\left(t_{2}\right)$, where $△{\bar{E}}_{evo}\left(0\to t_{0}\right)$, $△{\bar{E}}_{evo}\left(t_{0}\to t_{1}\right)$ and $△{\bar{E}}_{evo}\left(t_{1}\to t_{2}\right)$ can be derived from Equation (9), $△\bar{E}\left(t_{0}\right)$, $△\bar{E}\left(t_{1}\right)$ and $△\bar{E}\left(t_{2}\right)$ can be obtained from Equation (2).

## Appendix B. NSIT Conditions for Mixed State under Coherent Dynamics

According to ${\mathrm{NSIT}}_{(i)j}^{(n)m}$, ${\mathrm{NSIT}}_{(0)1,2}^{(n)m,l}$ and ${\mathrm{NSIT}}_{0(1)2}^{n(m)l}$, from Equations (3) to (5), and (13) to (18), we obtain the following quantities

$$P\left(Q^{+}\left(t_{1}\right)\right)-\sum_{\pm }P\left(Q^{\pm }\left(t_{0}\right),Q^{+}\left(t_{1}\right)\right)$$

$$=-[P\left(Q^{-}\left(t_{1}\right)\right)-\sum_{\pm }P\left(Q^{\pm }\left(t_{0}\right),Q^{-}\left(t_{1}\right)\right)]$$

$$=P\left(Q^{+}\left(t_{2}\right)\right)-\sum_{\pm }P\left(Q^{\pm }\left(t_{1}\right),Q^{+}\left(t_{2}\right)\right)$$

$$=-[P\left(Q^{-}\left(t_{2}\right)\right)-\sum_{\pm }P\left(Q^{\pm }\left(t_{1}\right),Q^{-}\left(t_{2}\right)\right)]$$

(A1) $$\begin{array}{c} =-\delta \sin^{2}\theta \cos\theta \sin^{2}\left(\frac{\omega \tau }{2}\right), \end{array}$$

$$P\left(Q^{+}\left(t_{2}\right)\right)-\sum_{\pm }P\left(Q^{\pm }\left(t_{0}\right),Q^{+}\left(t_{2}\right)\right)$$

$$=-[P\left(Q^{-}\left(t_{2}\right)\right)-\sum_{\pm }P\left(Q^{\pm }\left(t_{0}\right),Q^{-}\left(t_{2}\right)\right)]$$

(A2) $$\begin{array}{c} =-\delta \sin^{2}\theta \cos\theta \sin^{2}\omega \tau , \end{array}$$

$$P\left(Q^{+}\left(t_{1}\right),Q^{+}\left(t_{2}\right)\right)-\sum_{\pm }P\left(Q^{\pm }\left(t_{0}\right),Q^{+}\left(t_{1}\right),Q^{+}\left(t_{2}\right)\right)$$

$$=-[P\left(Q^{-}\left(t_{1}\right),Q^{-}\left(t_{2}\right)\right)-\sum_{\pm }P\left(Q^{\pm }\left(t_{0}\right),Q^{-}\left(t_{1}\right),Q^{-}\left(t_{2}\right)\right)]$$

(A3) $$\begin{array}{c} =-\frac{1}{4}\delta \sin^{2}\theta \cos\theta \sin^{2}\left(\frac{\omega \tau }{2}\right)\left(\cos2\theta +2\sin^{2}\theta \cos\omega \tau +3\right), \end{array}$$

$$P\left(Q^{-}\left(t_{1}\right),Q^{+}\left(t_{2}\right)\right)-\sum_{\pm }P\left(Q^{\pm }\left(t_{0}\right),Q^{-}\left(t_{1}\right),Q^{+}\left(t_{2}\right)\right)$$

$$=-[P\left(Q^{+}\left(t_{1}\right),Q^{-}\left(t_{2}\right)\right)-\sum_{\pm }P\left(Q^{\pm }\left(t_{0}\right),Q^{+}\left(t_{1}\right),Q^{-}\left(t_{2}\right)\right)]$$

(A4) $$\begin{array}{c} =\delta \sin^{4}\theta \cos\theta \sin^{4}\left(\frac{\omega \tau }{2}\right), \end{array}$$

$$P\left(Q^{+}\left(t_{0}\right),Q^{+}\left(t_{2}\right)\right)-\sum_{\pm }P\left(Q^{+}\left(t_{0}\right),Q^{\pm }\left(t_{1}\right),Q^{+}\left(t_{2}\right)\right)$$

$$=-[P\left(Q^{+}\left(t_{0}\right),Q^{-}\left(t_{2}\right)\right)-\sum_{\pm }P\left(Q^{+}\left(t_{0}\right),Q^{\pm }\left(t_{1}\right),Q^{-}\left(t_{2}\right)\right)]$$

(A5) $$\begin{array}{c} =\frac{1}{4}\sin^{2}\theta \left(\delta \cos\theta -1\right)\sin^{2}\left(\frac{\omega \tau }{2}\right)\left[2\sin^{2}\theta +\left(\cos2\theta +3\right)\cos\omega \tau \right], \end{array}$$

$$P\left(Q^{-}\left(t_{0}\right),Q^{+}\left(t_{2}\right)\right)-\sum_{\pm }P\left(Q^{-}\left(t_{0}\right),Q^{\pm }\left(t_{1}\right),Q^{+}\left(t_{2}\right)\right)$$

$$=-[P\left(Q^{-}\left(t_{0}\right),Q^{-}\left(t_{2}\right)\right)-\sum_{\pm }P\left(Q^{-}\left(t_{0}\right),Q^{\pm }\left(t_{1}\right),Q^{-}\left(t_{2}\right)\right)]$$

(A6) $$\begin{array}{c} =\frac{1}{4}\sin^{2}\theta \left(\delta \cos\theta +1\right)\left[2\sin^{2}\omega \tau -\sin^{2}\left(\frac{\omega \tau }{2}\right)\left(\cos2\theta +2\sin^{2}\theta \cos\omega \tau +3\right)\right]. \end{array}$$

Then, from Equations (A1) to (A6), we find that when one of the following conditions is satisfied: (1) θ=0; (2) $\tau =\frac{2\pi }{\omega }$; (3) $\theta =\frac{\pi }{2}$ and $\tau =\frac{\pi }{\omega }$, the NSIT conditions can not be violated, i.e., Equations (3) and (5) can not be violated.

## Appendix C. CNSIT Conditions for Mixed State under Coherent Dynamics

From Equations (1), (2), (6), (9), (13), (14) and (18), according to the two-time CNSIT conditions, i.e., $△E_{(i)j}^{(n),m}$ of Equation (6), $△E_{j}^{m}-\sum_{n}△E_{i,j}^{n,m}$ can be obtained as

$$△E_{1}^{+}-\sum_{\pm }△E_{0,1}^{\pm ,+}=△E_{2}^{+}-\sum_{\pm }△E_{1,2}^{\pm ,+}$$

(A7) $$\begin{array}{c} =\frac{\omega \cos\theta \left(\delta^{2}\cos2\theta -\delta^{2}-2\delta \cos\theta +\frac{2{\left(\delta \cos\theta -1\right)}^{2}}{\delta \cos^{3}\theta +\delta \sin^{2}\theta \cos\theta \cos\omega \tau -1}+2\right)}{4(\delta \cos\theta -1)}, \end{array}$$

$$△E_{1}^{-}-\sum_{\pm }△E_{0,1}^{\pm ,-}=△E_{2}^{-}-\sum_{\pm }△E_{1,2}^{\pm ,-}$$

(A8) $$\begin{array}{c} \frac{1}{2}\omega \cos\theta \left(\frac{1-\delta^{2}}{\delta \cos\theta +1}+\delta \cos\theta -\frac{\delta \cos\theta +1}{\delta \cos^{3}\theta +\delta \sin^{2}\theta \cos\theta \cos\omega \tau +1}\right), \end{array}$$

(A9) $$\begin{array}{c} △E_{2}^{+}-\sum_{\pm }△E_{0,2}^{\pm ,+}=\frac{\omega \cos\theta \left(\delta^{2}\cos2\theta -\delta^{2}-2\delta \cos\theta +\frac{2{\left(\delta \cos\theta -1\right)}^{2}}{\delta \cos^{3}\theta +\delta \sin^{2}\theta \cos\theta \cos\left(2\omega \tau \right)-1}+2\right)}{4(\delta \cos\theta -1)}, \end{array}$$

(A10) $$△E_{2}^{-}-\sum_{\pm }△E_{0,2}^{\pm ,-}=\frac{1}{2}\omega \cos\theta \left(\frac{1-\delta^{2}}{\delta \cos\theta +1}+\delta \cos\theta -\frac{\delta \cos\theta +1}{\delta \cos^{3}\theta +\delta \sin^{2}\theta \cos\theta \cos\left(2\omega \tau \right)+1}\right).$$

Then, according to the three-time CNSIT conditions $△E_{(0)1,2}^{(n)m,l}$ in Equation (7), from Equations (1), (2), (7), (9), (13), (14) and (18), $△E_{1,2}^{m,l}-\sum_{n}△E_{0,1,2}^{n,m,l}$ can be derived as

$$△E_{1,2}^{+,+}-\sum_{\pm }△E_{0,1,2}^{\pm ,+,+}=\frac{\delta \omega }{16\left(\delta \cos\theta -1\right)\left(\delta \cos^{3}\theta +\delta \sin^{2}\theta \cos\theta \cos\omega \tau -1\right)}\{-2\sin^{2}\theta \cos\theta$$

(A11) $$\times \left[\left(3\delta^{2}+4\right)\cos\theta +\delta \left(\delta \cos3\theta -2\cos2\theta -6\right)\right]+\sin^{2}\left(2\theta \right)\left(\delta^{2}\cos2\theta -\delta^{2}-2\delta \cos\theta +2\right)\cos\omega \tau \},$$

(A12) $$△E_{1,2}^{-,+}-\sum_{\pm }△E_{0,1,2}^{\pm ,-,+}=\frac{1}{2}\omega \cos\theta \left(\frac{1-\delta^{2}}{\delta \cos\theta +1}+\delta \cos\theta -\frac{\delta \cos\theta +1}{\delta \cos^{3}\theta +\delta \sin^{2}\theta \cos\theta \cos\omega \tau +1}\right),$$

(A13) $$△E_{1,2}^{+,-}-\sum_{\pm }△E_{0,1,2}^{\pm ,+,-}=\frac{\omega \cos\theta \left(\delta^{2}\cos2\theta -\delta^{2}-2\delta \cos\theta +\frac{2{\left(\delta \cos\theta -1\right)}^{2}}{\delta \cos^{3}\theta +\delta \sin^{2}\theta \cos\theta \cos\omega \tau -1}+2\right)}{4(\delta \cos\theta -1)},$$

$$△E_{1,2}^{-,-}-\sum_{\pm }△E_{0,1,2}^{\pm ,-,-}=\frac{\delta \omega }{16\left(\delta \cos\theta +1\right)\left(\delta \cos^{3}\theta +\delta \sin^{2}\theta \cos\theta \cos\omega \tau +1\right)}\{-2\cos\theta \sin^{2}\theta$$

(A14) $$\times \left[\left(3\delta^{2}+4\right)\cos\theta +\delta \left(\delta \cos3\theta +2\cos2\theta +6\right)\right]+\sin^{2}\left(2\theta \right)\left(\delta^{2}\cos2\theta -\delta^{2}+2\delta \cos\theta +2\right)\cos\omega \tau \}.$$

And according to $△E_{0(1),2}^{n(m)l}$ of Equation (8), from Equations (1), (2), (8), (9), (13), (14) and (18), $△E_{0,2}^{n,l}-\sum_{m}△E_{0,1,2}^{n,m,l}$ can be written as

$$△E_{0,2}^{+,+}-\sum_{\pm }△E_{0,1,2}^{+,\pm ,+}=\frac{\omega }{2}\{-\frac{1}{\cos\theta +\sin\theta \tan\theta \cos^{2}\omega \tau }+\cos\theta [\sin^{2}\theta \left(1-\cos\omega \tau \right)$$

(A15) $$+\frac{1+i}{\cos2\theta +2\sin^{2}\theta \cos\omega \tau +\left(1+2i\right)}+\frac{1-i}{\cos2\theta +2\sin^{2}\theta \cos\omega \tau +\left(1-2i\right)}]\},$$

(A16) $$\begin{array}{c} △E_{0,2}^{-,+}-\sum_{\pm }△E_{0,1,2}^{-,\pm ,+}=\frac{\omega \cos\theta {\left(\cos^{2}\theta +\sin^{2}\theta \cos\omega \tau \right)}^{2}}{\cos2\theta +2\sin^{2}\theta \cos\omega \tau +3}, \end{array}$$

(A17) $$\begin{array}{c} △E_{0,2}^{+,-}-\sum_{\pm }△E_{0,1,2}^{+,\pm ,-}=-\frac{\omega \cos\theta {\left(\cos^{2}\theta +\sin^{2}\theta \cos\omega \tau \right)}^{2}}{\cos2\theta +2\sin^{2}\theta \cos\omega \tau +3}, \end{array}$$

$$△E_{0,2}^{-,-}-\sum_{\pm }△E_{0,1,2}^{-,\pm ,-}=\frac{\omega }{2}\{\frac{1}{\cos\theta +\sin\theta \tan\theta \cos^{2}\omega \tau }+\cos\theta [\sin^{2}\theta \left(\cos\omega \tau -1\right)$$

(A18) $$-\frac{1+i}{\cos2\theta +2\sin^{2}\theta \cos\omega \tau +\left(1+2i\right)}-\frac{1-i}{\cos2\theta +2\sin^{2}\theta \cos\omega \tau +\left(1-2i\right)}]\}.$$

From Equations (A7) to (A18), we can find that the CNSIT conditions can not be violated (i.e., Equations (6) and (8) can not be violated), if one of the following conditions is satisfied: (1) θ=0; (2) $\theta =\frac{\pi }{2}$.

## Appendix D. ANSIT Conditions for Mixed State under Coherent Dynamics

For the ANSIT conditions, according to $△E_{(i)j}$ (Equation (10)), $△{\bar{E}}_{(0)1,2}$ (Equation (11)), and $△{\bar{E}}_{0(1)2}$ (Equation (12)), from Equations (2), (9) to (14), and (18), $△{\bar{E}}_{j}-△{\bar{E}}_{i,j}$, $△{\bar{E}}_{1,2}-△{\bar{E}}_{0,1,2}$, and $△{\bar{E}}_{0,2}-△{\bar{E}}_{0,1,2}$, can be obtained as

(A19) $$\begin{array}{c} △{\bar{E}}_{1}-△{\bar{E}}_{0,1}=△{\bar{E}}_{2}-△{\bar{E}}_{1,2}=-\delta \omega \sin^{2}\theta \cos^{2}\theta \sin^{2}\left(\frac{\omega \tau }{2}\right), \end{array}$$

(A20) $$\begin{array}{c} △{\bar{E}}_{2}-△{\bar{E}}_{0,2}=-\delta \omega \sin^{2}\theta \cos^{2}\theta \sin^{2}\omega \tau , \end{array}$$

(A21) $$\begin{array}{c} △{\bar{E}}_{1,2}-△{\bar{E}}_{0,1,2}=-\frac{1}{4}\delta \omega \sin^{2}\left(2\theta \right)\sin^{2}\left(\frac{\omega \tau }{2}\right)\left(\cos^{2}\theta +\sin^{2}\theta \cos\omega \tau \right), \end{array}$$

(A22) $$\begin{array}{c} △{\bar{E}}_{0,2}-△{\bar{E}}_{0,1,2}=\frac{1}{8}\delta \omega \sin^{2}\left(2\theta \right)\sin^{2}\left(\frac{\omega \tau }{2}\right)\left[2\sin^{2}\theta +\left(\cos2\theta +3\right)\cos\omega \tau \right]. \end{array}$$

It can be found from Equations (A19) to (A22) that if one of the following conditions is satisfied: (1) θ=0; (2) $\tau =\frac{2\pi }{\omega }$; (3) $\theta =\frac{\pi }{2}$; (4) δ=0, the ANSIT conditions can not be violated, i.e., Equations (10) and (12) can not be violated.
